# Particle Therapy to Overcome Cancer Radiation Resistance: “ARCHADE” Consortium Updates in Radiation Biology

**DOI:** 10.3390/cancers17091580

**Published:** 2025-05-06

**Authors:** Samuel Valable, Mathieu Césaire, Kilian Lecrosnier, Antoine Gilbert, Mihaela Tudor, Guillaume Vares, Dounia Houria Hamdi, Ousseynou Ben Diouf, Thao Nguyen Pham, Julie Coupey, Juliette Thariat, Paul Lesueur, Elodie Anne Pérès, Juliette Aury-Landas, Zacharenia Nikitaki, Siamak Haghdoost, Carine Laurent, Jean-Christophe Poully, Jacques Balosso, Myriam Bernaudin, Diana I. Savu, François Chevalier

**Affiliations:** 1 ARCHADE, Association for “Advanced Resource Center for Hadrontherapy in Europe”, 14000 Caen, France; samuel.valable@cnrs.fr (S.V.); mathieu.cesaire@hotmail.fr (M.C.); kilian.lecrosnier@ganil.fr (K.L.); antoine.gilbert@ganil.fr (A.G.); jthariat@gmail.com (J.T.); paul.lesueur89@gmail.com (P.L.); peres@cyceron.fr (E.A.P.); aurylandas@cyceron.fr (J.A.-L.); zacharenia.nikitaki@unicaen.fr (Z.N.); siamak.haghdoost@unicaen.fr (S.H.); carine.laurent@unicaen.fr (C.L.); poully@ganil.fr (J.-C.P.); jbalosso@chu-grenoble.fr (J.B.); bernaudin@cyceron.fr (M.B.); 2 Université de Caen Normandie, CNRS, Normandie Université, ISTCT UMR6030, GIP CYCERON, 14000 Caen, France; 3 Université de Caen Normandie, ENSICAEN, CNRS, CEA, Normandie Université, CIMAP UMR6252, 14000 Caen, France; hdh_jade@hotmail.com (D.H.H.); oben.diouf@univ-thies.sn (O.B.D.); 4 Department of Life and Environmental Physics, Horia Hulubei National Institute of Physics and Nuclear Engineering, 077125 Magurele, Romania; mihaela.tudor@nipne.ro; 5 Faculty of Biology, University of Bucharest, 030018 Bucharest, Romania; 6 Autorité de Sûreté Nucléaire et de Radioprotection (ASNR), PSE-SANTE/SESANE/LRTOX, 92260 Fontenay-aux-Roses, France; guillaume.vares@irsn.fr; 7 Mixed Research Exploration and Diagnosis (UMRED), UFR-Healthy, Iba Der THIAM University of Thies, Thies BP A967, Senegal; 8 Radiation Oncology Department, Centre François Baclesse, 14000 Caen, France; 9 Radiation Oncology Department, Centre Guillaume Le Conquérant, 76600 Le Havre, France; 10 Normandie University, UNICAEN, UNIROUEN, ABTE UR4651, Cancer Center François Baclesse, 14000 Caen, France

**Keywords:** hadrontherapy, radiation resistance, carbon ion therapy, hypoxia, cancer stem cells, radiosensitizers, normal tissues, bystander effect

## Abstract

Radiation therapy is a medical treatment that uses high doses of radiation to kill or damage cancer cells. It works by damaging the DNA within the cancer cells, ultimately causing cell death. Radiotherapy can be used as a primary treatment, adjuvant treatment in combination with surgery or chemotherapy or palliative treatment to relieve symptoms in advanced cancer stages. Radiation therapy is constantly improving in order to enhance the effect on cancer cells and reduce the side effects on healthy tissues. Our results clearly demonstrate that proton therapy and, even more, carbon ion therapy appear as promising alternatives to overcome the radioresistance of various tumors thanks to less dependency on oxygen and a better ability to kill cancer stem cells. Interestingly, hadrons also retain the advantages of radiosensitization approaches. These data confirm the great ability of hadrons to spare healthy tissue near the tumor via various mechanisms (reduced lymphopenia, bystander effect, etc.). Technology and machine improvements such as image-guided radiotherapy or particle therapies can improve treatment quality and efficacy (dose deposition and biological effect) in tumors while increasingly sparing healthy tissues. Radiation biology can help to understand how cancer cells resist radiation (hypoxia, DNA repair mechanisms, stem cell status, cell cycle position, etc.), how normal tissues may display sensitivity to radiation and how radiation effects can be increased with either radiosensitizers or accelerated particles. All these research topics are under investigation within the ARCHADE research community in France. By focusing on these areas, radiotherapy can become more effective, targeted and safe, enhancing the overall treatment experience and outcomes for cancer patients. Our goal is to provide biological evidence of the therapeutic advantages of hadrontherapy, according to the tumor characteristics. This article aims to give an updated view of our research in radiation biology within the frame of the French “ARCHADE association” and new perspectives on research and treatment with the C400 multi-ions accelerator prototype.

## Simple Summary

This work aims to present an overview of the complementary aspects of cancer treatment research using hadrontherapy performed in the French “ARCHADE” association over the past 15 years. The research ranges from the impact of hadrontherapy on cancer cells and its toxicity on normal tissue to the possibilities of radiosensitization with targeted drugs and, finally, to the non-targeted effects of high-LET radiations.

## 1. Introduction

The French hadrontherapy research association, named ARCHADE for “Advanced Resource Center for Hadrontherapy in Europe”, has several and complementary goals including fundamental and applied research in physics, biology and chemistry, and finally operational developments and clinical research, for the improvement of cancer treatment and imaging [1,2].

The work package 3 of the ARCHADE program is related to radiobiological data for hadrontherapy. This work package is based on close collaborations between three research laboratories in Caen, Normandy: ISTCT (UMR 6030), ABTE/ToxEMAC (UR 4651) and CIMAP/ARIA (UMR6252). Indeed, the city of Caen in the north west of France is a unique hotspot of hadronbiology research, thanks to the proximity of dedicated facilities such as GANIL (large accelerator of heavy particles, from carbon to uranium), Cyclhad (hadrontherapy center, with proton and soon with C-ions), and Cyceron (imaging platform) [3].

Within these research laboratories, various in vitro and in vivo experiments are performed with accelerated ions, particularly protons and C-ions. The goal of the present review article is to present the recent research progresses of the ARCHADE consortium, with a special focus on radiation biology.

Given the biophysical and biological characteristics of accelerated ions, radiotherapy with C-ions and even protons has the potential to improve local control and reduce normal tissue complications in the treatment of cancer patients, especially those with radio-resistant tumors [2]. However, fundamental research remains to be carried out before C-ion therapy achieves its promises. Even less is known about the patient’s safety, the interplay with co-morbidities, side effects or sequelae and the outcome of combined hadrontherapy with other therapies [4]. Combined therapies have been used in very few studies and, generally, chemotherapy demonstrated additive cytotoxicity when combined with C-ion therapy. It remains to be determined whether C-ions may set aside the need for radiosensitizing agents [1,2,5,6,7,8,9].

In the following paragraphs of this review article, the ARCHADE consortium aims at presenting unique and complementary aspects of cancer treatment with hadrontherapy (various cancer cell lines representing different tumor locations and several sources of radioresistance), with the impact on cancer cells, the toxicity on normal tissues, the possibilities of radiosensitization with targeted drugs and, finally, the non-targeted effects (bystander effect) of such irradiation conditions. The experimental conditions of these experiments are summarized in Table 1.

In the following section, the cut-off between low-LET and high-LET has been chosen at 50 keV/µm.

## 2. Radiobiology of Radioresistant Cancer Models: Advantages of Protons and C-Ions Relative to X-Rays

### 2.1. Determination of the RBE of Irradiations with Particles

#### 2.1.1. Chondrosarcoma (CHS) Models

A first study aimed to compare the effects of C-ions with various LET and X-rays on CHS cell lines cultured under in vitro regular conditions as a monolayer [10]. The goal was to investigate the radiosensitivity of these cell lines and assess RBE of high LET C-ions, particularly for X-rays resistant CHS cells. Four CHS cell lines were used: L835, CH2879, SW1353 and OUMS27. These cell lines included both X-ray-sensitive (L835, CH2879) and X-ray-resistant (SW1353, OUMS27) types. Three irradiation conditions were compared and X-rays (225 kV) were used as a reference irradiation method; C-ion irradiation (95 MeV/A) was performed with two different LET values, 28 keV/µm on native beam without degradation and 73 keV/µm using a degraded beam with polymethyl methacrylate (PMMA) absorber. In addition, cells were irradiated at the HIMAC facility in Japan, using a beam with an initial energy of 290 MeV/A and an average LET of 50 keV/µm for the micro-nucleus assays. The clonogenic survival assays showed varying radiosensitivity among the cell lines. As expected, high-LET C-ions (73 keV/µm) induced greater cell killing across all cell lines compared to X-rays and lower LET C-ions (28 keV/µm). RBE values for C-ions were significantly higher in X-ray-resistant cells. The SW1353 cell line showed prolonged G2 phase arrest and increased micronuclei formation after C-ions irradiation, indicating more severe DNA damage and repair complexity compared to X-rays. The study demonstrated the particular effectiveness of high-LET C-ions in killing CHS cells, especially those resistant to X-rays. The high RBE values observed (up to 5.4) suggest that C-ions could be a promising approach for treating CHSs, offering potential advantages over traditional X-ray therapy. However, further radiobiological studies are necessary to fully understand the mechanisms involved.

Following this initial study, a 3D CHS model was developed in order to validate and investigate the impact of different radiation qualities, especially low-LET and high-LET radiation, on CHS cells in an environment that mimics in vivo tumor microenvironment and tissue homeostasis (i.e., lower oxygen concentration and glucose availability in the core of the 3D structure, more complex matrix) after therapeutic radiation exposure [11]. Thus, the next study used this 3D culture model (3DCM) with SW1353 CHS cells. The cells were exposed to low-LET radiation (X-rays) and high-LET radiation (^18^O ions, LET = 103 keV/μm) at GANIL; this high-LET was used since it was in the range of LETs of clinical C-ion SOBP [34]. The study observed differential cellular responses between low-LET and high-LET radiation in terms of proliferation and DNA damage and repair. High-LET irradiation resulted in higher and persistent gamma-H2AX induction compared to low-LET irradiation. The responses to radiation of the 3D model differed significantly from those of the 2D culture, particularly for high-LET exposure. This 3D model, which mimics the in vivo microenvironment, seems to be more relevant for studying radiation effects. The 3D model showed potential as a validation tool for evaluating the RBE of therapeutic protons or ion beams by measuring the variation in RBE along the depth-dose profile of these beams. The study concluded that the 3D physioxic CHS model is a valuable tool for studying the impact of radiation quality on CHS cells. It demonstrated that high-LET radiation has a more pronounced biological effect compared to low-LET radiation in this model. The 3DCM could be used for future investigations of radiation quality impacts on normal cartilage and mesenchymal stem cells (MSCs), as well as for understanding the radiation resistance mechanisms of CHS in clinical settings.

#### 2.1.2. Glioblastoma (GB) Models

The RBE depends on the biological model, the measured endpoint and its magnitude and the particle type and its energy level. Although many studies showed that the RBE of C-ions increases with LET in many types of solid tumors, only a few addressed this issue in GB. Thus, the biological response of GB cells to C-ion irradiation was first investigated at LET values ranging from 28 to 100 keV/µm to approximate those used in the clinics [12].

In this study, two human adherent GB cell lines (U251 and GL15) were irradiated with X-rays or C-ions. At 24 h after seeding, GB cells were exposed at room temperature to X-rays at doses ranging from 0 to 8 Gy (X-Rad 225Cx, Precision X-ray Inc, CYCERON platform, France). X-rays were delivered at a mean energy of 80 keV at a dose rate of 2 Gy/min (voltage: 225 kV, current: 13 mA, Cu filter: 1 mm). For C-ion treatment, GB cells were exposed to C-ion beam (^12^C) with physical doses ranging 0 to 6 Gy (IRABAT D1, GANIL, Caen, France) at the energy of 95 MeV/A [35]. To generate C-ions with various LET, a degrader based on an increasing thickness of PMMA was placed upstream of the culture flasks, e.g., 28 keV/µm (PMMA = 0), 50 keV/µm (PMMA = 13.9 mm) and 100 keV/µm (PMMA = 17.9 mm). To confirm the delivered doses for each culture flask, dose deposit maps of C-ions were generated by an ionization chamber-based dosimetry (DOSION III, LPC laboratory, Caen, France) [36]. From clonogenic survival assays, RBE was calculated for each individual cell line as the ratio of D37 X-rays/D37 C-ions (D37 is the dose giving 37% of survival).

GB cells were more sensitive to C-ion irradiation than to conventional X-ray irradiation at all LETs, as evidenced by survival curves and radiobiological parameter measurements. The RBE was 1.3, 1.8 and 2.9 for LET values of 28, 50 and 100 keV/µm, respectively. In comparison to photons, the exposure of U251 cells to C-ions at the high-LET value (100 keV/µm) led to four times lower cell survival at 2 Gy. The experimental data evidenced that RBE was strongly, linearly and positively correlated to LET (r^2^ = 0.99), confirming that U251 GB cell sensitivity to C-ion irradiation is a function of LET. In parallel, the effects of C-ions according to LET values on the cell cycle were evaluated by flow cytometry. From the cell cycle profiles, the RBE increase was associated with a delay in G2/M arrest at low-LET (28 keV/µm) and with a prominent G2/M blockage at high-LET (100 keV/µm). These data highlighted that the biological effectiveness of C-ion irradiation on GB cells results in a LET-dependent G2/M arrest, followed by GB cell accumulation in the subG1 phase. In summary, this in vitro study demonstrated for both GB cell lines (U251 and GL15) that RBE is strongly correlated with LET (ranging from 28 keV/µm to 100 keV/µm) and RBE dependence on LET is associated with higher cell cycle changes, particularly on G2/M arrest.

### 2.2. Impact of Hypoxia on Radioresistance

#### 2.2.1. GB Models

Hypoxia, defined as the reduction of tissue oxygen levels, is a major feature of human solid tumors, including GB [37]. Hypoxia correlates with poor patient outcome, aggressive tumor phenotype and resistance to chemotherapy and radiotherapy in GB. Tumor hypoxia is known to limit the efficacy of ionizing radiations, a concept called “oxygen effect” measured by the oxygen enhancement ratio (OER). OER values above one demonstrated hypoxia induced radioresistance. The OER depends on physical factors such as pO_2_ and LET. Previous in vitro studies with low-LET radiation have shown that cancer cells are 2–3 times more radioresistant under hypoxic compared to normoxic conditions, whereas OER can reach the value of one with high-LET radiation. Interestingly, OER is inversely correlated with LET, suggesting a potential clinical advantage of high-LET radiotherapy with ion beams such as C-ions compared to low-LET photon or proton irradiations. In addition to these physicochemical mechanisms of radiations, biological parameters may affect C-ion efficiency such as the cellular adaptive responses to hypoxia mediated by the activation of the hypoxia-inducible transcriptional factor-1 (HIF-1). HIF-1 has been suggested to be involved in radioresistance under hypoxic conditions by acting on multiple cellular pathways (i.e., metabolism, cell cycle, DNA repair pathways, apoptosis, maintenance of cancer cell stemness). Studies also showed that HIF-1 inhibition sensitizes GB cells to X-rays. Furthermore, the involvement of hypoxia in radioresistance has also been indirectly demonstrated by the modulation of HIF-dependent gene expression such as EPO (erythropoietin) [38], which is also involved in tumor progression [39]. We aimed to evaluate in vitro whether high-LET particles, especially C-ion irradiation, can overcome the contribution of hypoxia to radioresistance, and whether HIF-dependent genes, such as erythropoietin (EPO), could influence GB sensitivity to C-ion exposure [12]. In this study, the two human GB cells (U251, GL15) cultured in normoxic (21% O_2_) or hypoxic conditions (1% O_2_) were exposed to X-rays or to C-ion beams with different LET (28, 50 and 100 keV/µm), and for complementary experiments, genetically modified U251 GB cells with downregulated EPO signaling by EPO receptor inhibition (EPOR) were used. Cell survival, radiobiological parameters, cell cycle and ERK pathway activation were assessed under these conditions. Twenty-four hours after seeding, GB cells were exposed at room temperature to X-rays (X-Rad 225Cx) or C-ion beam (^12^C, IRABAT D1, GANIL).

**Figure 1:**
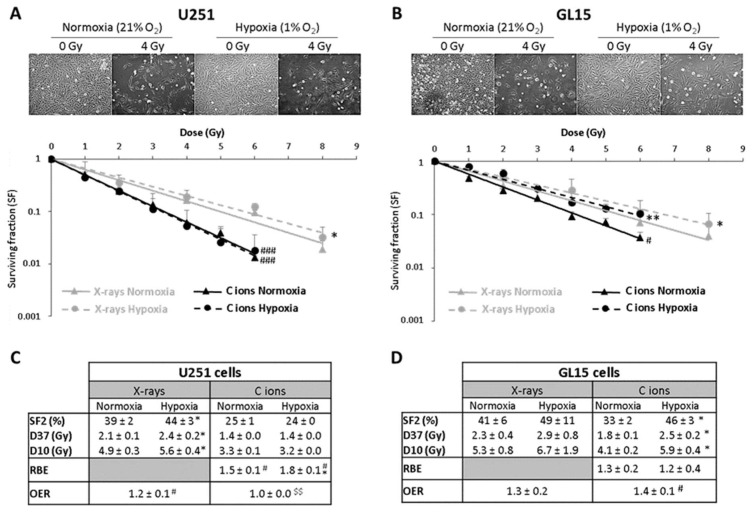
Hypoxia induces a GB cell type-dependent radioresistance to C-ion irradiation; reprinted with permission from [12], Copyright 2020MDPI. (**A**,**B**) Representative photographs of the cell morphology observed 72 h after C-ion irradiation in normoxia or hypoxia (4 Gy, C-ions 28 keV/µm) for U251 cells ((**A**) **top part**) and GL15 cells ((**B**) **top part**). Survival curves from clonogenic assays performed in normoxic (21% O_2_) or hypoxic conditions (1% O_2_) after X-rays or C-ions (28 keV/µm) for U251 cells ((**A**) **down part**) and GL15 cells ((**B**)**-down part**). Fisher’s LSD post-hoc test after a significant two-way ANOVA (group and dose effects): * *p* < 0.05, ** *p* < 0.01 vs. normoxia for X-rays or C-ion irradiation; # *p* < 0.05, ### *p* < 0.0001 vs. X-rays in normoxia or hypoxia. (**C**) Quantification of radiobiological parameters obtained from X-rays or C-ion irradiations for U251 cells and (**D**) GL15 cells grown in normoxic or hypoxic conditions. For SF2, D37 and D10: * *p* < 0.05 vs. normoxia for X-rays or C-ion irradiation (Fisher’s LSD post-hoc test after a significant one-way ANOVA). For RBE: # *p* < 0.05 vs. theoretical value = 1 (univariate t-test) and * *p* < 0.05 vs. normoxia (Student’s *t*-test). For OER: # *p* < 0.05 vs. theoretical value = 1 (univariate *t*-test) and $$ *p* < 0.01 vs. X-rays (Student’s *t*-test). All data of the figure are presented by mean ± SD; all experiments performed in triplicate (*n* = 3) for both irradiation and oxygen conditions.

The results obtained from clonogenic survival assays evidenced that hypoxia induces a radioresistance of U251 cells for X-rays (OER = 1.2) but not for C-ions (OER = 1.0). Unlike U251 cells, GL15 cells exhibited also radioresistance to C-ions under hypoxia (OER X-rays = 1.3 and OER C-ions = 1.4) (Figure 1).

To understand the potential underlying mechanisms of the differential response to C-ion irradiation of these two GB cell lines under hypoxia, the ERK pathway known to be involved in hypoxia-induced radioresistance to X-rays and to be modulated by C-ions was investigated [13]. By western-blotting approach, C-ion irradiation did not modulate ERK phosphorylation under either oxygenation conditions in U251 cells. In contrast, C-ion irradiation under hypoxia (1% O_2_) induced a significant increase in ERK activation in GL15 cells. These results suggest that hypoxia-induced radioresistance in C-ion irradiation context is cell-type dependent and might be partly explained by differences in ERK activation.

Furthermore, this study also assessed the involvement of HIF-1 in GB sensitivity to C-ions, by focusing on HIF-1-dependent genes such as EPO. The inhibition of EPOR expression on U251 cells led to decreased cell survival compared to scrambled shRNA cells after exposure to C-ion irradiation. These results indicate that EPOR knockdown radiosensitizes U251 cells to C-ions by increasing apoptosis consecutive to an increase of DNA damage.

Collectively, all the results of this in vitro study performed on human GB cells demonstrate that high-LET values of C-ion beams overcome hypoxia-induced radioresistance but that it is dependent on the cell type and the activation status of the ERK signaling pathway. In addition, these results underscore the importance of the EPO signaling pathway, a HIF target gene, in optimizing the response to C-ions radiotherapy.

#### 2.2.2. Lung Cancer Cells Models

In several studies, the differential effects of X-ray and C-ion radiations were also investigated on A549 non-small-cell lung cancer (NSCLC) cells radioresistance under normoxic (20% O_2_) and hypoxic (1% O_2_) conditions [14,15]. Gene expression, cell cycle modulation and NF-κB-dependent transcriptional response were analyzed. The main goal was to understand the mechanisms underlying radioresistance induced by hypoxia, focusing on the PI3K/AKT and NF-κB target genes signaling pathways and their roles in cell survival and cell cycle response following radiation-induced DNA damage. Hypoxic conditions alone enhanced processes related to cell migration and motility, suggesting increased aggressiveness. X-ray irradiation under hypoxia further enhanced cell migration and motility, while C-ion irradiation did not show such enhancement. An upregulation of PI3K/AKT pathway target genes was observed under hypoxia, both with and without irradiation, a significant upregulation of DNA repair and apoptosis genes, with notable differences between the radiation types. Five NF-κB target genes were upregulated following irradiation, regardless of oxygenation status or radiation type. The genes FAS and CDKN1A showed the highest upregulation. Cytokine secretion of IL-6 and IL-8 was increased under chronic hypoxia and irradiation. IL-6 secretion peaked 6 h after irradiation, while IL-8 peaked 24 h after. Both cytokines enhance tumor propagation through immune evasion, angiogenesis and metastatic potential. Hypoxia induced radioresistance to X-rays, potentially through epithelial–mesenchymal transition (EMT) mechanisms, which were absent with C-ion irradiation. CDKN1A gene upregulation was observed across all conditions, linking cell cycle arrest and repair processes to the PI3K/AKT pathway. Both low- and high-LET radiation increase NF-κB-mediated mRNA transcription and cytokine release in hypoxic A549 NSCLC cells, potentially enhancing cancer cell survival and propagation. The study concluded that hypoxia-induced radioresistance in A549 cells is linked to the PI3K/AKT signaling pathway, which promotes cell survival, proliferation and migration. X-ray radiation under hypoxia exacerbates these effects, whereas C-ion radiation is less influenced by hypoxic conditions. The transcriptional response of NF-κB target genes is greater following C-ion exposure than X-ray irradiation. X-rays upregulate more NF-κB responsive genes involved in oncogenic transformation and cancer cell survival compared to C-ions. These findings highlight the potential for targeting the PI3K/AKT and NF-κB pathways to overcome hypoxia-induced radioresistance in NSCLC and suggest C-ion therapy as a promising alternative due to its reduced dependency on oxygenation status for efficacy.

### 2.3. The Impact of CSCs on Radioresistance

#### 2.3.1. GB Models

The highest expectations of ions irradiations rely on their better RBE compared with X-ray and their potential capacity to destroy (CSCs). Glioblastoma stem cells (GSCs) are a specific subpopulation of GB cells with properties of tumor stem cells, such as unlimited proliferation, self-renewal, differentiation and metastatic abilities; they also have high DNA repair capacity, which explains a major part of the GB radioresistance. We wondered if C-ion irradiation alone, thanks to its radiobiological advantage, could overcome GB radioresistance. In 2018 [16], the impact of C-ion irradiation on two GSCs lines (R633, TG1) was explored. The impact of C-ions was evaluated by using the limiting dilution assay, which allows an estimation of stem-like cell frequency. Indeed, the analysis of the frequency of sphere-forming cells (gliospheres), a surrogate property of brain cancer stem-like cells, was used to estimate the stem like cell frequency. Irradiations were performed on the IRABAT beam line from GANIL and the CATANA beam line from the Instituto Nazionale di Fisica Nucleare—Laboratori Nazionali del Sud (Catania, Italy) with C-ions (^12^C) having a LET evaluated at 50 keV/μm. Surprisingly, for both cell lines, a single irradiation of 2 Gy C-ions physical dose (assuming a RBE of around 2) was less effective than 4 Gy X-rays in decreasing GSC fractions.

As reported above, hadrontherapy, in particular, C-ion irradiation, is of great interest to target GB cells [12]. The greater sensitivity of GB to C-ions could also be explained by the absence of HIF-1 activation after irradiation in contrast to X-rays. However, some cells remained resistant to C-ions, and we postulated that the remaining, radioresistant cells could be GSCs. GSCs are suspected to be the most radioresistant cells due to their quiescent state and their high efficacy in DNA repair. The increase in the GSC pool could result from the ability of cancer cells to revert into a stem-like phenotype, a process known as cell reprogramming (or dedifferentiation), and, thus, contribute to tumor recurrence. This cell reprogramming can be promoted by the stabilization of HIF transcription factors or X-ray irradiation. However, the impact of hadrontherapy on cancer cell reprogramming is poorly documented. The ARCHADE consortium is currently investigating the impact of particle therapy (protons or C-ions) in combination with the inhibition of HIF transcription factors on radiation-induced stemness [40] with the hypothesis that these two therapeutic strategies will act synergistically to disable GB reprogramming and, thus, GSC-induced GB recurrence.

The methodology used is as follows. The human GB cell line U-87MG was used. Adherent cells were exposed to 2 or 4 Gy C-ions (95 MeV/A) with LET 50 keV/µm. For protons, the cells were exposed to 4 or 8 Gy with LET 4.7 keV/µm. X-rays (4 or 8 Gy) were used as a reference irradiation method. Irradiations were performed at GANIL (IRABAT), CYCLHAD (Proteus One IBA) and CYCERON (X-RAD 225Cx PXI), for C-ions, protons and X-rays, respectively. The cells were treated with pharmacologic inhibitors, specific of HIF-2 (PT-2385) or targeting both HIF-1 and HIF-2 (2-methoxyestradiol). After irradiation and HIF inhibition, the formation of gliospheres (non-adherent cells) was monitored in order to evaluate the ability of GB cells to reprogram into GSCs. GB cells were then detached and re-seeded in a stem cell adapted medium supplemented with EGF and bFGF cytokines. Then, molecular and cellular responses to treatments (irradiation +/− HIF inhibition), including stemness properties, were characterized by complementary methodologies. These preliminary results show that protons and C-ions decreased the formation of gliospheres contrary to X-rays.

#### 2.3.2. CHS Models

The goal of the study was to explore the effectiveness of a multimodal treatment strategy combining C-ion irradiation, miRNA-34 and an mTOR inhibitor in targeting CSCs in high-grade CHS [17]. This approach aimed to overcome the resistance of CHS to conventional therapies by targeting the CSCs. The biological model includes high-grade CHS cell lines and tumor xenografts in mice. The irradiation conditions involve the exposure to C-ion beams; the treatment also included miRNA-34, a tumor suppressor miRNA that targets multiple oncogenic pathways, and rapamycin, an mTOR inhibitor, which is used to disrupt the mTOR signaling pathway commonly upregulated in cancer cells. The results demonstrated the existence of a radioresistant CHSALDH+ CSCs subpopulation, suppressed by miRNA-34. C-ion irradiation alone was not sufficient to suppress CSCs. miRNA-34 triggered the loss of CSCs, probably via its target gene KLF4, and protected against tumor formation in mouse xenografts. Furthermore, the inhibition of mTOR by rapamycin targeted CSCs via FOXO3 and miRNA-34. Altogether, the results suggested that the association of mTOR inhibition by rapamycin with miRNA-34, as an experimental treatment, may be able to overcome the CSCs-associated radioresistance of CHS exposed to C-ion therapy.

### 2.4. Radiosensitization of Resistant Cancer Models with Targeted Drug: Combination with Protons or C-Ions Versus X-Rays

#### 2.4.1. PARP Inhibitor on CHS

New promising therapies targeting DNA damage repair pathways have recently emerged, such as PARP inhibitors (PARPis). PARPis inhibit the action of poly (ADP-ribose) polymerases (PARPs), proteins that detect damaged DNA and then activate DNA repair signaling pathways. The aim of the study was to compare the effectiveness of combining PARPi and irradiation (X-ray, proton or C-ion irradiations) in the CHS cell line (CH2879 cells) in vitro [18]. CH2879 cells were exposed to the PARPi olaparib two hours before the irradiations. PARPi induced a radiosenstizing effect in CH2879 cells with X-rays, proton and C-ion irradiations at an 1.3, 1.8 and 1.5 enhancement ratio, respectively, in clonogenic survival assays. The combination of PARPi with any type of irradiation also reduced cell proliferation. The radiosensitivity of CH2879 cells is associated with mutations in homologous recombination repair genes, such as RAD50, SMARCA2 and NBN. This study demonstrated the capacity of the PARPi olaparib to radiosensitize CHS cells to X-ray, proton and C-ion irradiation.

Another study, involving IDH wild type and IDH mutant CHS cell lines (OUMS27, JJ012) without homologous recombination deficiency phenotype, showed that olaparib significantly sensitized CHS cells to low LET X-ray but not to high LET C-ions [19]. Cell lines with IDH mutations showed increased radiosensitivity with olaparib combined with X-ray, indicating a potential link between IDH status and treatment efficacy. High-LET radiation, such as C-ions (73 keV/µm), independently induced substantial cell death, with a threefold higher biological efficiency than X-rays. Olaparib was effective in inhibiting PARylation but led to an increased PARP-1 expression in certain cells, which may counteract its radiosensitizing effects. In addition, DNA repair pathways, including non-homologous end joining (NHEJ), were activated in response to high LET radiation, which diminished the effects of PARP inhibition. Finally, normal chondrocytes also responded to olaparib with X-ray radiation, suggesting potential risks to healthy tissue if not carefully targeted. Overall, combining olaparib with low-LET radiation could enhance radiosensitivity in IDH-mutant CHS, though precision in targeting is crucial to protect healthy tissue.

#### 2.4.2. PARP Inhibitor on GB

Several mechanisms have been implicated in GB radioresistance including cell cycle, tumor microenvironment such as hypoxia, metabolic alteration, GSCs, tumor heterogeneity, microRNAs and DNA damage and repair. In fact, GB cells, and particularly GSCs, exhibit highly proficient DNA repair mechanisms [9]. We recently showed that GB radioresistance could be related with an important dynamic and changing pattern of phosphorylation proteome forms [41], such as BRCA1, MDC1, H2AX and TP53BP1 in the early stages after radiation.

In 2018, three PARPi (olaparib, talazoparib and AG14361) were evaluated in vitro as radiosensitizers, combined with X-ray or C-ion irradiation for GSCs (TG1 and R633) [16]. The irradiations were performed on the IRABAT beam line of GANIL and the CATANA beam line of Catania with C-ions (^12^C) with LET evaluated at 50 keV/μm. Different types of irradiations (X-rays or C-ions) were combined with temozolomide +/− talazoparib, olaparib or AG14361 and their impact on the reduction in GSCs after irradiation was analyzed. The results were compared to the reference schedule: X-ray irradiation combined with temozolomide. The use of PARPi combined with X-rays and even more C-ion irradiation drastically reduced the GSC frequency of GB cell lines in vitro. Finally, the combination of talazoparib with C-ions appeared to be the most promising schedule to reduce the GSC. For example, the decrease in the TG1 GSC fraction was 2.8- or 3.5-fold greater when talazoparib was combined with 2 Gy C-ions than with 4 Gy X-ray irradiation. Aside from DSBs, high-LET irradiations induce more complex DNA damage than photons, called oxidative clustered DNA lesions (OCDLs). These DNA lesions are repaired mainly by BER, in which PARP plays a major role. In the presence of talazoparib, these sublethal damages are not repaired and could lead to lethal damages, explaining the higher benefit from the association of PARPi with high-LET C-ion irradiation.

A recent study investigated whether ATR and ATM inhibition enhances interferon (IFN) signaling in GB cells subjected to irradiation with X-rays, protons and C-ions [20]. It aimed to explore the potential of combining ATR inhibitors with high-LET particle irradiation to enhance cancer cell radiosensitivity and antitumor immune signaling. Human GB cell lines (U-251 and T98G) were irradiated with X-rays (low-LET, ≈4 keV/µm), protons with LETs of 4.8 keV/µm (low-LET) and 41.9 keV/µm (high-LET) and C-ions with LETs of 28 keV/µm and 73 keV/µm. ATR and ATM inhibitors were applied in conjunction with irradiation. High-LET protons and C-ions induced stronger DNA damage responses compared to low-LET protons and X-rays. ATR inhibition abrogated G2 checkpoint arrest and significantly increased type 1 IFN signaling across all irradiation modalities, with the strongest effect observed with high-LET particles. Both inhibitors enhanced radiosensitivity, but ATR inhibition produced a greater increase in IFN-β secretion, particularly with high-LET irradiation. ATM inhibition also increased IFN signaling but less effectively than ATR inhibition. Enhanced signaling was attributed to mechanisms like micronucleus formation and cytosolic DNA exposure triggering the cGAS-STING pathway. The study demonstrated that ATR inhibition enhances IFN signaling in GB cells following irradiation with X-rays, protons and carbon ions. The effect was dependent on the LET of the particles, with higher LET correlating with stronger IFN responses. These findings suggest that combining ATR inhibitors with particle therapy could potentiate tumor radiosensitization and antitumor immune effects, potentially improving the efficacy of radiotherapy for GM.

#### 2.4.3. Inhibitors of Proliferation: KRAS G12C Inhibitor on Lung Cancer

Lung cancers, in the localized and inoperable stage, are currently treated with X-ray radiotherapy and chemotherapy but survival remains poor (43% at 5 years). Some radioresistant and chemoresistant mechanisms in lung cancer are related to KRAS mutations.

KRAS mutations are the most common mutations in lung cancers (25–37% in lung adenocarcinoma). KRAS mutations induced mutated KRAS proteins structurally in active form, leading to continuous cancer cells proliferation. Targeted therapies such as Sotorasib—a tyrosine kinase inhibitor—selectively binding KRAS G12C mutated proteins have been reported to be effective in KRAS G12C mutated lung cancer patients with good tumor responses and better progression-free survival of patients. Combining these KRAS G12C inhibitors and irradiation could enhance efficacy in KRAS G12C mutated lung cancer [42]. Another mechanism of radioresistance in NSCLC is related to cancer stem cells (CSC) that could initiate a tumor repopulation after X-rays irradiation and form metastasis. C-ion irradiation could eradicate CSC thanks to the higher RBE.

The aim of this study is to determine how sotorasib could radiosensitize cancer cells and how combining C-ion irradiation and sotorasib could eradicate CSC in the KRAS G12C mutated cell line (H358 cells) in vitro. Experiments are ongoing with survival and proliferation assays to study the radiosensitization effect and tumorspheres assays and RTq PCR on CSC markers to study the impact on CSC.

#### 2.4.4. Nanoparticles with a CHS Model

**Figure 2:**
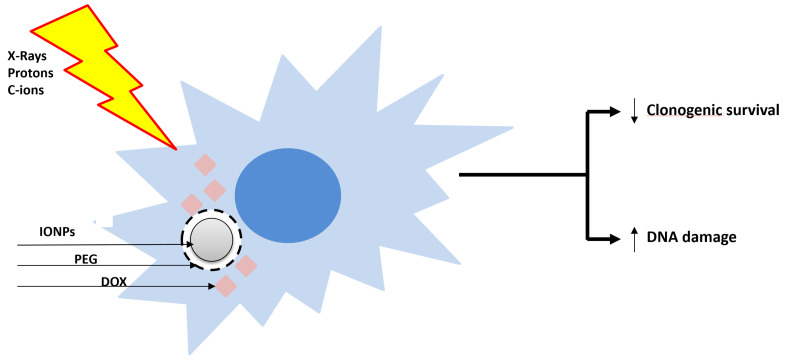
Sensitization of radioresistant CHS cells with the core–shell doxorubicin-loaded nanoparticles and X-ray, proton and C-ion radiations. Core–shell iron oxide (Fe_3_O_4_) nanoparticles (IONP) were encapsulated in polyethylene glycol (PEG) and loaded with doxorubicin (DOX). ↑: increase; ↓: decrease.

The use of nanoparticles (NPs) targeting the tumor in combination with accelerated charged particles is an emerging promising therapeutic strategy. Two recent works highlighted, for the first time, the ability of the core–shell iron oxide (Fe_3_O_4_) nanoparticles (IONP) encapsulated in polyethylene glycol (PEG) loaded with doxorubicin (IONP_DOX_) to sensitize SW1353 CHS cells to high-energy protons (155 MeV, LET 1.6 keV/µm), low-energy protons (18 MeV, LET 12.6 keV/µm), C-ions (95 MeV, 73 keV/µm) and X-ray irradiation [21,22]. IONPs have been used as a doxorubicin vehicle [43] due to their magnetic transport ability, biocompatibility for healthy tissue and ability to enhance the irradiation effect by generating reactive oxygen species. In addition, the controlled delivery of doxorubicin is expected to induce cyto- and genotoxicity inside the tumor, while sparing the healthy tissues (Figure 2). Indeed, CHS cells loaded with NPs and then irradiated either with X-rays, C-ions or protons exhibited an enhanced reduction in clonogenic survival as compared to the cells exposed to irradiation alone. The calculated values of dose modifying factors (DMF) for 0.1 survival fraction sustained these results, their values being higher than 1. The NPs generated the highest sensitization effect of CHS cells to low-LET protons irradiation (DMF_SF=0.1_ = 2.011 ± 0.118) as compared to carbon ions (DMF_SF=0.1_ = 1.2 ± 0.1), high-LET protons (DMF_SF=0.1_ = 1.098 ± 0.272) and X-rays (DMF_SF=0.1_ = 1.05 ± 0.03) irradiation. Additionally, the combined effect of NPs with all the types of irradiations caused a higher number of DNA lesions measured as micronuclei than the irradiation alone. Therefore, we suggested that the accumulation of DNA damage contributes to the cytotoxicity mechanisms of the combined treatments.

## 3. Radiotoxicity of Protons and C-Ions Relative to X-Rays on Normal Tissues

### 3.1. Radiation Side Effects on Healthy Brain

#### 3.1.1. Cognitive Deficits and Fatigue After Brain Irradiation

Radiation therapy (RT) using X-rays RT is performed annually on several hundreds of thousands of patients with brain tumors. Although it undoubtedly improves patient survival, brain RT inevitably leads to adverse effects due to irradiation of the healthy brain tissue. Radiation toxicities appear a few days to a few years after RT and lead to cognitive decline and fatigue occurring, permanent cognitive disabilities associated with a reduction in the quality of life of long-surviving patients. To improve the management of radiation-induced brain injury, it is essential to better understand the pathophysiological mechanisms underlying radiation adverse effects, and also to identify non-invasive biomarkers to detect brain damage early and predict cognitive deficits. To address these issues, the use of animal models is necessary. Thus, our consortium has recently developed and characterized a radiation-induced brain injury in the adult rat profiling the general health status, cognitive alterations and brain damage. Based on a multiparametric and longitudinal characterization of a whole-brain irradiation (WBI) rat model, the aim of this preclinical study was to define magnetic resonance imaging (MRI) biomarkers to predict brain damage underlying cognitive decline [44,45].

Adult male Wistar rats (6 months old) were exposed to a fractionated 30 Gy whole-brain irradiation (WBI: 3 × 10 Gy for 3 consecutive days) with the X-RAD 225Cx irradiator dedicated to small animals. To ensure spatial accuracy and homogeneity of the deposited dose in the brain, WBI was planned with a treatment planning system (TPS) (SmART-Plan software, Precision X-Ray, Madison, CT, USA) and the actual doses absorbed by the brain tissue were measured by in vivo dosimetry based on a scintillating fiber dosimeter [46]. A longitudinal study was conducted in acute (days to weeks), early (1 to 3 months) and late (4 to 6 months) phases after WBI with complementary approaches. A battery of behavioral tests was performed at the BRP platform (Behavioral Research Platform, Unicaen, France) to quantify fatigue (homemade test and open field) and cognitive impairments (object recognition test and passive avoidance task). In parallel, sequential MRI analyses (7T Bruker, Cyceron Platform) were undertaken at different times after WBI to quantify brain volumetry, brain vascularization with cerebral blood volume (CBV) measurement and tissue integrity from diffusion tensor imaging (DTI). To confirm brain damage identified by MRI, immunohistochemical analyzes (IHC) were performed to analyze blood vessels, astrocytes, microglia/macrophages and white matter fibers.

Significant animal fatigue and a locomotor activity reduction from the first weeks after WBI and in the late phase after WBI were reported. As observed in patients, short-term memory deficits were evidenced early, whereas long-term memory is altered several months after WBI. Behavioral impairments were associated to vascular damage and microstructure modifications such as astrogliosis, microglia/macrophages activation and white-matter degeneration observed by IHC. The MRI results showed that the irradiation schedule used in this study did not induce any necrosis or edema visible on T2-weighted imaging. However, in the chronic phase, irradiated rats displayed a significant brain atrophy and reduced CBV as well as alterations of the brain tissue microstructure. Interestingly, the analysis of mean diffusivity parameter based on diffusion imaging revealed significant early brain tissue modifications observed from 2 weeks after WBI [44,45]. Recently, the consortium investigated the ability of a computational approach to the analysis of anatomical MRI images, called deformation-based morphometry (DBM), to determine, in a completely objective manner, the brain structures most vulnerable to irradiation. In the irradiation-induced brain injury model (WBI, 30 Gy) developed in adult rats, DBM analysis performed on T2w MRI images highlighted local macrostructural changes over time, some of which were transient and others long lasting after brain irradiation. DBM revealed two vulnerable brain areas, namely, the corpus callosum and the cortex [45]. Finally, in this animal model of cerebral radiotoxicity, multiparametric MRI was relevant to detect early and late radiation-induced brain injury. In future investigations, blood samples collected in this animal model will be used to assess cytokines/molecules markers to monitor toxicity induced by brain RT.

Although the mechanisms underlying RT-induced cognitive decline have been extensively discussed, the etiology of cancer-related fatigue remains poorly understood. In some clinical studies based on different cancer types, skeletal muscle loss was described after cancer treatment and recently, it has been evidenced that sarcopenia, defined as a decrease of muscle mass and physical performance, is a factor of poor prognosis after RT. From the WBI model in rats (30 Gy), muscle MRI, behavioral tests evaluating fatigue and locomotion and histological studies demonstrated, for the first time at the preclinical scale, that brain irradiation induces muscle damage (muscle atrophy and change in muscle fiber typology) that is associated with radiation-induced fatigue. It was also shown that physical activity improves animal survival, reduces muscle damage and decreases fatigue [44].

To minimize the adverse effects of brain RT, the current strategies are mainly based on the optimization of photon therapy ballistics with new RT modalities such as intensity modulated radiation therapy (IMRT), volumetric intensity-modulated arc therapy (VMAT), stereotactic radiotherapy (SRT) and stereotactic radiosurgery (SRS) in order to reduce the doses to organs at risk as much as possible. Ion beam therapy, especially proton and C-ion radiotherapy, seems relevant to attenuate irradiation of healthy brain tissue since it offers a better ballistic accuracy thanks to the Bragg peak. In this context, the animal model of radiation brain injury was refined by reducing the irradiation brain volume [47] or by modulating beam orientation for WBI to spare organs at risk [48]. As expected, in accordance with the results obtained with brain tumor patients treated by RT, the refinement of irradiation protocols mitigates brain damage and cognitive deficits in the rat. In future investigations, the consortium will use the animal models based on brain irradiation with X-rays as a reference to determine the short- and long-term effects of hadrontherapy (protons and C-ions) on healthy brain tissue.

#### 3.1.2. Systemic Inflammation After Brain Irradiation

Leucocytes are part of the immune system, which protects the body against foreign invaders, as well as cancer. Leucocytes represent a heterogeneous population, with subpopulations in both lymphoid and myeloid lineages originating from their common hematopoietic stem cells within the bone marrow that exert an essential impact on tumor regulation. Both leucocyte lineages participate to cancer surveillance.

In recent years, it has been proposed that radiotherapy may have stimulatory or suppressive immune effects at the tumor and tissue levels, including blood. Of these effects, the role of immunosuppressive effects of fractionated RT, such as radiation-induced lymphopenia (RIL), are now well reported. However, until recently, the suppressive effect of radiotherapy on the immune system has been largely ignored in routine practice. Radiation-induced immune suppression was investigated in the 1970s, at the time of the premises of immunotherapy, then forgotten for about 30 years until a recent rebound in interest with the onset of new immunotherapy modalities. RIL may occur even when lymphoid organs are not present in the irradiated zone, such as in the case of brain irradiation. In humans, recovery from RIL can take months to years. Moreover, RIL is associated with poor overall survival in patients with solid tumors, including brain tumors. Thanks to the improvement of radiotherapy technologies, a more focused dose delivery can be achieved. For instance, the use of protons instead of conventional radiotherapy using X-rays can spare normal tissue with a smaller entrance dose, no exit dose and small lateral beam penumbra, which could translate into a better therapeutic ratio. Proton therapy is performed in various brain tumors and has shown less severe RIL compared to X-rays.

However, the different effects of protons on myeloid cells and lymphocyte subpopulations have not yet been sufficiently studied. A protocol was recently developed, firstly aiming to investigate the differences between X-ray and proton irradiations under different radiation conditions on circulating leucocyte subpopulations in rodent. Statistical regression models were applied to investigate the relationships between radiation parameters and physiology-based parameters and their impact on leucocyte subpopulations. In addition, previous literature has hypothesized that RIL is a result of the direct radiation exposure of circulating lymphocytes during the irradiation period, i.e., direct cell-killing effect. To investigate the direct lymphocyte-killing effect hypothesis, the radiation dose to which lymphocytes in the blood are exposed was estimated during the irradiation period, as was whether other organs could be involved. Radiation-induced lymphopenia occurred after X-ray but not proton brain irradiation in lymphoid subpopulations (T-CD4+, T-CD8+ and B and NK-cells). There was an increase in neutrophil counts following protons but not X-rays. Monocytes remained unchanged under both X-rays and protons. Besides the irradiation particle type, the irradiated volume and dose rate had a significant impact on NK-cell, neutrophil and monocyte counts but not on T-CD4+, T-CD8+ and B-cells. The irradiation of the blood had a heterogeneous impact on leucocyte subpopulations: Neutrophil counts remained stable with increasing dose to the blood, while lymphocyte counts decreased with increasing dose (T-CD8+-cells > T-CD4+-cells > B-cells > NK-cells). The direct cell-killing effect of the irradiation dose on the blood mildly contributed to radiation-induced lymphopenia. Lymph node exposure significantly contributed to lymphopenia and partially explained the distinct impact of irradiation type particles on circulating lymphocytes [23]. Based on these data, brain irradiation with X-rays and protons exerted different effects on systemic inflammation. According to the X-ray results, the ALC (absolute lymphocyte count) and LMR (lymphocyte-to-monocyte ratio) decreased, and the ALC but not the LMR recovered to baseline after irradiation. Both X-rays and protons increased the NLR (neutrophil-to-lymphocyte ratio) during irradiation, which recovered after protons but not X-rays. Both the irradiation volume and dose rate had a pronounced effect on the NLR. The interplay of the leukocyte subpopulation was observed under X-ray and proton irradiations, with normalization of the proton group by day 28 [24]. To further a deeper investigation of the hypothesis of direct lymphocyte killing by in vivo irradiation, several studies have aimed to model this effect by (1) modeling the dose that each lymphocyte receives during irradiation and (2) modeling the dose effect on lymphocytes (i.e., lymphocyte radiosensitivity). The linear–quadratic model, commonly used to analyze the impact of radiation on cells, has shown limitations when applied to lymphocytes. Under these considerations, a new model, the saturation model, was proposed in an attempt to outperform the LQ model. The time dependency and differences in lymphocyte survival under exposure to X-rays or protons using LQ and saturation models were further investigated. The saturation model considers a negative exponential relationship between radiation dose and cell response to radiation, addressing potential non-linear responses of lymphocytes to radiation. It offers a more accurate representation of the lymphocyte response to radiation. The saturation model can be used to assess T-lymphocyte survival following exposure to X-rays and protons and observe time dependencies [49].

### 3.2. Radiation Side-Effects on the Cartilage

The study aimed to assess senescence induction in human cartilage as a direct response to C-ion irradiation in the context of CHS treatment, compared with X-rays [25]. The focus was on using human articular chondrocytes (HACs) cultured in physioxic (which means for cartilage a low O_2_ pressure) as 3D cartilage model (3DCaM) and comparing it to a 2D classic culture model as a control. Radiation-induced senescence was investigated because it has been suggested as a potential mechanism to explain low-LET-induced cartilage attrition and osteoarthritis in irradiated patients. HACs in both 2D and 3DCaM were irradiated in physioxia (2% O_2_), C-ion irradiations were performed using 75 to 95 MeV/A C-ions (LET~33 keV/µm) at GANIL with a dose rate of 1 Gy/min and X-ray irradiations were conducted using the X-RAD 225 Cx (225 kV, 13 mA) with a dose rate of 2 Gy/min. In the 2D culture, C-ions were found to be more effective to induce senescence compared to X-rays, with a RBE of 2.6. In the 3DCaM, there was no significant difference in senescence induction between C-ions and X-rays. Both irradiation types induced senescence in approximately 8.3% of the samples. The study showed that the 3D culture conferred relative radioresistance compared to the 2D culture, possibly due to factors like chromatin modification, adhesion, apical/basal orientation, mechanical tension and soluble factor diffusion. The study concluded that C-ions do not induce more senescence in the 3D cartilage model than X-rays, suggesting that C-ions are not more toxic than X-rays for articular cartilage in case of radiotherapy.

### 3.3. Radiation Side-Effects on the Skin

To compare the toxicity of C-ions versus photons in skin, in vitro studies on normal human skin fibroblasts were performed [26,27]. The irradiation conditions were as close as possible to the reality of skin irradiation by clinical radiotherapy. The cells were exposed to radiation at confluence stage. For X-rays, a clinical machine of the radiotherapy department of Cancer Centre François Baclesse (Caen, France) was used (5 MV photons). Concerning C-ions, the irradiations were performed at GANIL with C-ions of 72 MeV/A and a LET of 33.6 keV/µm corresponding to the LET of the entrance plateau upstream from the SOBP. Survival experiments showed that SF2 and D_0_ were strongly decreased by a factor of 9.5 and 4.6 by C-ions compared to X-rays, respectively. Moreover, RBEs were high and similar to those published for cancer cells in the literature with 4.8 at D_0_ and 3.3 at D10% and the α/β ratio was increased by a factor 83 after C-ions compared to X-rays. To better understand the mechanisms leading to these results, biomarkers of genotoxicity, oxidative stress and inflammation were measured and compared between C-ions and X-rays at irradiation doses corresponding either to D_0_ or to D10%. DNA damage biomarkers were less or equally increased immediately after C-ion irradiation compared to X-rays, but they were increased at later times up to 2 weeks after irradiation concerning excreted 8-oxodG and micronuclei. This suggested that DNA repair could play a major role after exposure to C-ions or that secondary products of DNA damages may appear at later times. Moreover, lipid peroxidation and inflammatory cytokines were increased and antioxidant enzyme activities were decreased after C-ions compared to X-rays up to 3 weeks after irradiation. Indeed, RBE calculation for all these parameters showed that carbon ions compared to X-rays led to a decrease in cell survival but also in antioxidant capacity, whereas they led to an increase in DNA damage and DNA repair delay, an increase in lipid and protein oxidation and an increase in oxidative status and inflammatory cytokines (Table 2).

An important question arises regarding the differences of normal tissue dose delivery when using scattered versus scanned proton beams. To address this question, in vivo studies on C57Bl/6 mice were performed to compare the two modalities of proton beam delivery [28]. Mice were whole-body irradiated in the protontherapy center of Curie Institute (Orsay, France) using the C230 IBA accelerator in the SOBP at an energy of 190.6 MeV. Dose rates for double scattering (DS) and pencil beam scanning (PBS) were 8 Gy in 15 s and 8 Gy in 3.3 min (2.4 Gy/min), respectively, corresponding to the standard rate used in patient treatments. Survival curves showed no difference between DS and PBS delivery. However, DNA damage in lymphocytes was 4.3-fold higher after PBS than after DS even 3 months after the sublethal dose of 7.5 Gy. Concerning oxidative stress, the results differ according to the organs. Concerning inflammation, it seems that there was a trend to an increase in plasmatic IL-1α and TNF-α after PBS compared to DS. A transcriptomic study on the skin of these mice showed completely different profiles: Only one gene was common in the differentially expressed genes [29]. Non-coding RNA was also studied and only PBS led to 14 differentially expressed ncRNA with 49 potential mRNA-ncRNA interactions. In general, these two studies showed that 3 months after proton irradiation, DNA damage, oxidative stress and inflammation pathways were not returned to basal level in normal tissues.

### 3.4. Radiation Side-Effects on the Stem Cells: Focus on the Role of Oxidative Stress in the Response of Normal Adipose-Derived Stem Cells

Expanding the efforts to elucidate the molecular mechanisms underlying oxidative stress and cellular responses to ionizing radiation [30,50,51], the role of Nrf2 signaling in the survival and stemness of human adipose-derived stem cells (ADSC) was investigated [31]. These cells, known for their regenerative capacity and differentiation potential, represent an important model for studying radiation-induced damage and repair mechanisms. The focus was on how oxidative stress, modulated by the Nrf2 pathway, impacts ADSCs survival and differentiation after exposure to three different radiation qualities: X-rays (CellRad^®^ Faxitron irradiator, Cyceron Platform, set to 125 kV, 4.7 mA, 0.3 mm copper filter, 2 Gy/min.), protons (ProteusOne^®^ IBA, at CYCLHAD SAS, Caen, into a 2 cm SOBP with energy range between 110 MeV to 129 MeV), and C-ions (IRABAT, GANIL, 95 MeV/A; degraded to a LET~33 keV/µm). To probe the role of Nrf2, the inhibitor ML385 was used, which significantly reduced the expression of Nrf2-dependent proteins HO-1 and NQO1, confirming its functionality. ADSCs were exposed to increasing doses of radiation (0–4 Gy) to examine survival dynamics and differentiation into adipogenic and osteogenic lineages. C-ions exhibited the highest cytotoxicity (LD50 Carbon: 1.61 Gy), followed by protons (LD50 proton: 2.23 Gy) and X-rays (LD50 X-rays: 3.5 Gy), reflecting the greater RBE of ion radiotherapy. Pretreatment with ML385 sensitized cells to all radiation qualities, with the strongest effect observed for X-rays (33% reduction in LD50), highlighting a radiation-quality-dependent role for Nrf2 in modulating cellular resistance.

Radiation also negatively impacted the differentiation potential of surviving ADSCs. To this end, the DI50 metric was used. This parameter represents the dose of radiation that inhibits differentiation by 50%. X-rays led to the greatest inhibition of adipogenesis (DI50: 2.69 Gy) and osteogenesis (DI50: 1.79 Gy), while C-ions and protons preserved the differentiation capacity to a greater extent. The addition of ML385 further impaired differentiation across all radiation qualities, reducing adipogenic and osteogenic differentiation by 36% and 29%, respectively. These findings suggest that particle radiotherapy, particularly protons and C-ions, may better preserve stem cell functionality and regenerative potential compared to conventional X-rays. The study underscores the critical role of Nrf2 in regulating cellular responses to radiation-induced oxidative stress. By modulating ROS levels and supporting antioxidant defenses, Nrf2 preserves the stemness and differentiation capacity of ADSCs under normal conditions [52]. However, Nrf2 inhibition amplifies radiation sensitivity by reducing cellular defenses, particularly under X-ray exposure, where the oxidative damage pathway is more pronounced. This dual role highlights the therapeutic potential of selectively targeting Nrf2 in clinical contexts, balancing the protection of normal tissues with the radiosensitization of tumors. These findings also emphasize the potential advantages of particle radiotherapy over traditional X-rays, particularly for applications in long-term cancer survivors and pediatric patients. Protons and C-ions not only induce complex DNA damage more effectively in tumors but also reduce off-target effects in normal tissues, including stem cells, due to their unique energy deposition profiles (e.g., Bragg peak). These results contribute to a growing body of evidence supporting the integration of particle radiotherapy in treatment protocols, particularly in cases where preserving tissue regeneration is paramount. In conclusion, this work highlights the importance of understanding the molecular interplay between radiation quality, oxidative stress and stem cell function.

## 4. Non-Targeted Effects of High LET Particles (C-Ions and Protons) Compared to X-Rays: Radiation Induced Bystander Effects

### 4.1. Analysis of Bystander Effects

The effects of radiation on CHS cells [32] were investigated, focusing on both direct effects (locally directly occurred by irradiation) and bystander effects induced by X-rays and C-ions (in cells not targeted by the irradiation). Specifically, it assessed the impact on cell survival, DNA damage and the secretion of bystander factors that influence the proliferation and viability of neighboring non-irradiated cells.

This study used SW1353 CHS cells for direct irradiation experiments and T/C-28a2 chondrocyte cells for bystander effect experiments. Cells were irradiated with X-rays and C-ions (95 MeV/A; LET~73 keV/µm) at various doses. The study found that C-ions resulted in a lower survival fraction of directly irradiated CHS cells compared to X-ray irradiation. This indicated higher effectiveness of C-ions in killing cancer cells; RBE values of 2.49 and 3.58 were calculated using D10 and D37 values, respectively.

Conditioned medium from irradiated SW1353 cells negatively affected the proliferation and survival of non-irradiated T/C-28a2 chondrocyte cells. This bystander effect was mediated through factors secreted by the irradiated cells, which were able to cause DNA damages and to reduce cell viability in the non-irradiated cells. These effects were assessed through clonogenic assays, micronuclei formation and real-time cell analysis using impedancemetry.

This study concluded that C-ion irradiation is more effective than X-rays in directly targeting CHS cells due to its higher LET. However, both types of radiation induce significant bystander effects that can impair the proliferation and viability of neighboring non-irradiated chondrocytes. These findings highlight the possible relevance of considering both direct and bystander effects in radiotherapy to minimize damage to healthy tissues surrounding the tumor.

### 4.2. Analysis of Bystander Factors Secreted by CHS Cells

The intercellular communication between irradiated CHS cells and bystander chondrocytes was analyzed using proteomics approaches [53]. Secretome analysis of the conditioned medium from low-dose irradiated CHS cells revealed significant insights into the protein compositions and their roles. These approaches allowed us to propose new bystander mediator candidates and specific cellular responses potentially involved in these non-targeted effects.

Comparative analysis between irradiated (0.1 Gy) and control conditions (0 Gy) revealed 87 significantly modulated protein groups, with 55 more abundant and 32 less abundant proteins in the conditioned medium of irradiated cells. Notably, polyadenylate-binding protein 1 (P11940) was over-secreted 23.8 times in irradiated cells. Additionally, several ribosomal proteins (e.g., 60S ribosomal protein L34, 40S ribosomal protein S6) and proteins involved in oxidative response, cell migration and DNA damage response (e.g., Acetyl-CoA acetyltransferase, Septin-7, E3 ubiquitin-protein ligase RBBP6) were significantly increased. The presence of these proteins, especially those related to stress granules and oxidative responses, suggests their involvement in radiation-induced bystander effects. Stress granules, known to form under stress to protect cells, were unexpectedly found in the conditioned medium of irradiated cells. This novel finding suggests a potential role in intercellular communication, previously undocumented in bystander effect studies.

Proteins such as glyoxalase domain-containing protein 4 and endoplasmic reticulum aminopeptidase 1, typically associated with exosomes, were also enriched. This supports the hypothesis that exosome-mediated signaling could be a key mechanism in the bystander effect [2]. Further, the quantification of 8-oxo-dG in the conditioned medium indicated an increased oxidative stress level in irradiated cells, which likely contributes to the bystander effect. The conditioned medium also significantly impacted the motility of bystander chondrocytes, increasing from 5 to 13 h post-treatment, aligning with observed changes in proteins related to cell junction and adhesion (e.g., actin, desmoplakin).

Western blot validation confirmed the modulation of key proteins (cyclophilin A, thioredoxin, alpha-enolase, RPLP0, HSC70, HSPA9 and CCT3) in bystander cells, corroborating the proteomic findings. Cyclophilin A and thioredoxin, involved in interleukin signaling and cell redox homeostasis, respectively, were particularly noteworthy. Thioredoxin’s role in regulating transcription factors mediating responses to environmental stress, including radiation, was highlighted, further linking oxidative stress to bystander effects.

The identification of stress granules and specific exosome-related proteins provides new avenues for understanding the molecular mechanisms underlying radiation-induced bystander effects.

In conclusion, these proteomic analyses identified key proteins modulated by low-dose irradiation, highlighting their potential roles in bystander signaling. These findings contribute to the growing understanding of the complex intercellular communication processes involved in radiation-induced bystander effects and suggest new biomarker candidates for further investigation.

In a second study, we evaluated the bystander effects in non-irradiated chondrocytes that received conditioned medium from CHS cells irradiated with X-rays and C-ions [33]. Specifically, it sought to understand how different irradiation qualities and doses influence the proteome of bystander chondrocytes and the secretion of inflammatory cytokines in human vascular endothelial cells (HUVEC).

SW1353 CHS cells were irradiated with X-rays and C-ions (95 MeV/A; LET~73 keV/µm) at doses of 0.1 and 2 Gy. The conditioned media from irradiated cells were then transferred to the non-irradiated T/C-28a2 chondrocytes and HUVECs. A deep proteomic analysis was performed on recipient cells.

In bystander chondrocytes exposed to X-ray generated conditioned media, significant changes were observed in the protein translation pathway, IL-12 pathway and oxidative stress pathway. In bystander chondrocytes exposed to C-ion-generated conditioned media, the G1/S pathway and mitotic G2 DNA damage checkpoint pathway were notably engaged. Both low-dose X-ray and C-ion irradiations altered the regulation of chromosome separation. Stress granules were significantly enriched, indicating their possible role in reducing cellular metabolism to protect proteins. HUVECs showed variations in inflammatory cytokine secretion when exposed to conditioned media from irradiated CHS cells.

The study provided insights into the specific pathways and mechanisms involved in the bystander response of chondrocytes to different qualities and doses of irradiations.

The bystander effects are dose- and radiation-quality-dependent, with distinct pathways being activated in response to X-rays and C-ions, including antioxidant activity (specific to X-rays) or PTEN activity (specific to C-ions). This research may suggest potential new mechanisms and therapeutic targets related to the bystander effects in the tumor microenvironment during radiotherapy.

### 4.3. Bystander Activity of Matrikines from Collagen Irradiation

The study aimed to explore the presence of a bystander effect between irradiated and non-irradiated chondrocytes at low radiation doses, and to investigate whether this bystander effect is due to the formation of “matrikines” following degradation of the extracellular matrix (ECM) of cartilage by X-rays or C-ion beams [54]. Since collagen is the main constituent of the ECM, it is assumed that these matrikines are fragments of collagen coming from the direct effect of ionizing radiations on these macromelecules. The study seeks to propose a list of collagen “matrikine” peptides with various cellular activity by examining their in vitro toxicity against chondrocytes.

First, it is shown that chondrocytes synthesize an ECM in vitro. Chondrocyte cells T/C-28a2 were irradiated at low density in 25 cm^2^ flasks, with sham irradiation controls for comparison. Direct and bystander effects of irradiations were assayed by clonogenic survival.

A significant bystander effect was observed between irradiated and non-irradiated chondrocytes at low radiation doses (around 0.1 Gy).

Second, fragments of collagen due to direct radiation effects are identified thanks to an innovative strategy using collagen mimetic peptides. These peptides were irradiated in the gas phase by ionizing radiation (X-rays and C-ions) but also UV light, and fragmentation was analyzed using mass spectrometry [55].

**Figure 3:**
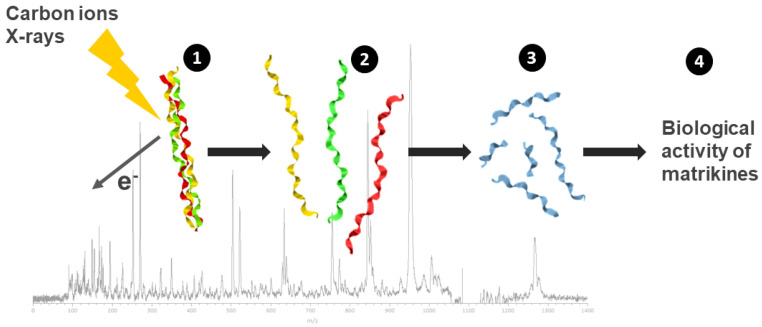
Analysis of the direct effects of radiation on the structure, stability and biological activity of collagen-fragmentation peptides. Collagen mimetic peptides, with a triple-helical structure were submitted to ionizing radiation (C-ions and X-rays) in gas-phase condition (1). Following triple-helix dissociation (2), chain fragmentation and small peptide formation (Pro–Pro–Gly) were observed (3) with a preferential cleavage sites on the glycine–proline peptidic bond. The resulting peptides from radiation-induced collagen-fragmentation were analyzed on chondrocytes culture and displayed a matrikine-like biological activity (4).

The glycine–proline (Gly–Pro) peptide bond in collagen was identified as a highly preferential cleavage site due to direct radiation effects, independent from the nature of the radiation (Figure 3).

Third, from the collagen sequence, 46 peptides resulting from Gly–Pro cleavage of collagen were selected and tested for toxicity on chondrocytes in vitro. The study then proposed a list of collagen peptides with varied cellular activity, which might be matrikines.

The study concludes that ionizing radiation induces a bystander effect between irradiated and non-irradiated chondrocytes at low doses, possibly due to the release of matrikines formed by the cleavage of collagen Gly–Pro peptide bonds following X-ray or C-ion irradiation of the ECM. This understanding can help in predicting and mitigating side effects of radiotherapy, such as local inflammation and fibrosis, by targeting specific collagen peptides that influence cellular activity.

## 5. Discussion

Particle therapy is gaining increasing recognition in oncology due to its superior dosimetric and biological advantages over conventional X-ray radiotherapy [56,57]. It is primarily indicated for radioresistant, rare, recurrent and radio-induced tumors—collectively known as the “4Rs” of hadrontherapy [58]. These include malignancies that demonstrate poor response to conventional radiation, such as sarcomas, adenoid cystic carcinoma, mucosal melanoma and certain gliomas. Additionally, it offers a viable treatment option for patients experiencing tumor recurrence after prior radiation therapy, where further X-ray may pose unacceptable toxicity risks. Furthermore, its application in radio-induced malignancies—secondary tumors arising from previous radiotherapy—presents a promising approach for managing these challenging cases. The biological mechanisms underlying particle therapy’s efficacy are related to its ability to induce complex DNA damages, which are less likely to be repaired by cancer cells, and its potential to enhance antitumor immune responses.

CHS is a rare and often aggressive malignant bone tumor known for its intrinsic resistance to conventional radiotherapy. This resistance is primarily attributed to its hypoxic microenvironment, low cell proliferation rate and upregulated DNA damage repair mechanisms [8]. Several studies have shown that C-ion radiations were more efficient in inducing complex DNA damage, leading to enhanced cancer cell death in CHS [59,60]. Radiation biology studies on CHS have demonstrated that C-ions induce prolonged G2/M cell cycle arrest and persistent DNA double-strand breaks [10,60]. Preclinical investigations further suggest that C-ions may modulate the tumor microenvironment by reducing angiogenesis and cancer stem cell activation; both are implicated in treatment resistance and recurrence [61]. On the other hand, radiation biology studies have shown a significant potential for CHS cells to propagate the stress signal from irradiation to non-irradiated-bystander cells located in the close environment. High-LET irradiated CHS cells induced significant DNA damage in co-cultured bystander fibroblasts [62]. Using a medium transfer protocol, these observations were confirmed with low doses of C-ions. The observations included a reduced survival rate, an increase in micronuclei formation in the bystander chondrocytes and elevated levels of cytokines such as TNF-α and IL-6 in the bystander conditioned medium [32,33,53].

As observed with CHS, GB are tumors that are very resistant to low-LET particles; in addition, they have an intrinsic resistance mediated by various mutations, the presence of stem cells and extrinsic parameters such as a pronounced hypoxia that also participate in the resistance and the recurrence. Targeting hypoxic cells with high LET particles was suggested many years ago, with the first clinical evidence published in 2006 [63]. Thereafter, Tinganelli et al., [64] validated a model describing the OER dependence versus pO2 and LET, validating the added value of using high-LET particles to reduce OER. In this study, oxygen pressure is suggested as a main factor of resistance and our ARCHADE consortium also confirmed this result in other human GBM cell lines. However, we and others also introduced other factors that could influence the response to high LET particles for a given oxic situation. Differences in intracellular pathways could provide some clues in line with published data showing that HIF1 but also ROS distribution could explain the greater effect of C-ions relative to X-Rays [65]. The role of stem cells or even the ability of radiation therapy to favor the dedifferentiation into stem cells are hypothesized and we demonstrated that GSC could even survive to the presence of C-ions [12,16]. Therefore, being able to further target the CSC will remain a challenge in the future. It also raises the question of addressing patient heterogeneity and further characterizing the tumors thanks to multi-omics approaches.

Targeting aggressive brain tumor remains a quite challenging issue. The diagnosis occurs often late, discovering tumors of large volume with already migrating invasive cells. Therefore, the clinical target volume (CTV) is often large, resulting in side effects. For instance, the link between irradiated volume and lymphopenia is something that needs to be further understood. The consortium recently reported the link between brain irradiation and radiation-induced lymphopenia. Using proton beam brain irradiation, less RIL was observed compared to using photons. Interestingly, recent data demonstrate in vitro, that for a given dose, protons kill more lymphocytes than photons [66]. This suggest that the conservative effect of hadron on lymphocyte could rather come from ballistic properties, as reviewed in [67] and recently discussed in a Kaplan lecture [68]. A decreased volume of irradiation has also other benefits regarding the other effects of irradiation. In this review, we report the effect of photons on micro- and macrostructure changes in the brain that are associated with changes in cognition. As a perspective, our ARCHADE consortium is currently conducting clinical trials to address the effect of protons versus photons in brain [69,70]. In the present study, a link between brain irradiation and fatigue observed in rodents was reported and linked with remote effects on skeletal muscles. In the future, we also believe that decreasing the volume of irradiation with particle therapy will also reduce the side effects mediated by photons. However, considering both ballistic properties and RBE, one should further address the side effects of ion beam therapy after brain irradiation.

The use of particle radiotherapy is currently expanding, especially for the radioresistant tumors [71,72,73,74,75,76], but despite some improvement of local control, the challenge to enhance the therapeutic effectiveness remains. The combination of particle therapy with radiosensitizers is recommended for improving radioresistant tumor management [77,78,79,80]. Tumor sensitization is a key component in developing novel innovative therapeutic approaches. DNA damage repair PARP inhibitors had the potential to radiosensitize CHS cells towards X-ray, proton and C-ion irradiation and also GSCs towards X-ray and C-ion irradiation [16]. The sensitizing effect was generally greater for charged particles. In agreement with our studies, various previous works indicated that the most effective radiosensitization was obtained with PARPi in combination with particle beams [81,82,83,84,85,86,87]. Interestingly, in our recent study [20], ATR and ATM inhibitors not only sensitized GB cells exposed to X-ray and particle (protons and C-ions) irradiation but also enhanced IFN-β secretion in a LET-dependent manner, the highest level being found in cells co-treated with ATR inhibitor and high-LET C-ions. The ATR inhibitor abrogated, while ATM prolonged, G2 arrest, a result consistent with other studies using X-ray irradiation [88,89,90]. Recent studies showed also the sensitizing potential of ATR and ATM inhibitors of cells exposed to high-LET hadron irradiation [91,92]. These studies suggest that DNA repair inhibitors in combination with particle irradiation may be more appropriate than those in combination with conventional X-ray irradiation to induce a tumor-specific radiosensitization and anti-tumor immune response. Due to the lack of exit particles, it is expected that these effects will be reduced in the surrounding normal tissues. Further investigations of combining DNA damage repair inhibitors with other agents as immune checkpoint blockade along with radiotherapy may be performed.

Nanoparticle-based drug delivery approaches may be considered as a strategy to improve targeting the tumors and enhance the efficacy of radiation therapy while minimizing side effects [93]. Two recent works proved that iron oxide NPs (IONPs) as doxorubicin carriers used in combination with X-ray, proton and C-ion irradiation amplified CHS cell death [21,22]. A few other studies showed the ability of IONPs to enhance cancer cell death effect of X-ray and ion-beam irradiation [94,95,96,97,98,99,100]. All the radiosensitizers described in our studies seem to enhance the efficacy of particle therapy, and in the future, more validation studies need to be performed to speed up clinical applications.

The biological effects of hadrons on healthy tissues have been poorly studied, in particular according to the modality of dose delivery or in comparison with photons. Indeed, normal tissue irradiation, according to the modality of dose delivery (scattered vs. scanned beams), could result in differences regarding oxidative stress, DNA damage or inflammation leading to side effects. In this way, the irradiation effects on organs at risk, such as the heart or lungs, need to be investigated. The skin is also one of the organs most at risk as, for instance, regarding breast irradiation by X-rays, more than 50% of the patients will encounter acute skin side effects and up to 50% late skin side effects [101]. Our studies on the biological effects of C-ions on normal skin cells [26,27] showed that macromolecular damage was strongly increased and that antioxidant defense was decreased during the first weeks after C-ion irradiation versus X-ray irradiation. These results could explain late skin side effects previously reported [102]. Concerning the side effects of protons on normal tissues, one of the main issues is the dose delivery modality, which is very different when beams are scattered or scanned. In our studies [28,29], we pointed out some specific biomarkers that could be correlated to clinical outcomes such as radio-necrosis observed in pediatric patients treated for brain tumors [103] but also to physical data (for example, LET maps). All together, these studies pointed out that particular attention should be paid to normal tissues surrounding high-LET particle irradiated tumors, focusing on normal tissue specificity and to dose delivery modalities.

## 6. Conclusions

These results demonstrate the ability of “ARCHADE consortium” to lead increasingly collaborative research addressing innovative and fundamental themes such as stem cell radiobiology, in-depth investigation of bystander phenomena, research into intrinsic or extrinsic (hypoxia) determinants of tumor radioresistance, the evolution of anti-tumor immunity in the tumor microenvironment, pioneering exploration of the toxicology of macromolecular fragmentation products, pharmacological-irradiation combinations interacting with DNA damage repair pathways, etc.

Overall, these approaches are part of a progression that will continue, with questions that will become increasingly clinical thanks to the development of therapeutic activities. This will require the gradual introduction of research on animal models, which will be able to build solidly on this fundamental foundation. Thus, by relying on in vitro and in vivo approaches, future directions in radiobiology research should focus on (i) understanding radioresistance mechanisms, particularly, determining which ones can be overcome by hadrontherapy; (ii) in-depth exploration of early and late toxicity of healthy tissues according to age (pediatrics versus adults) and organs, which represents a significant problem for the application of hadrontherapy, particularly with regard to aseptic osteonecrosis of the face and sacrum; (iii) interactions with anti-tumor immunity; (iv) modeling and analysis of individual determinants of radiation-induced cancer risk or promotion of tumor dissemination according to the type of irradiation; etc.

### Perspectives

The main objective of ARCHADE is to establish a European center for training, research and development in preclinical and clinical hadrontherapy. This center, within the CYCLHAD Society, aims to provide beam time for research centers and institutes, clinics and industrial companies on a national and international scale [3]. CYCLHAD ProteusOne^®^ system, a single-room compact proton therapy equipment delivering 250 MeV protons by active scanning, has been treating patients since July 2018 and available for experimental irradiations since December 2020. In the same place, the upcoming C400 superconductive cyclotron will deliver beams of light ions (protons to oxygen ions, including helium and C-ions) for therapeutic and research purposes. Set to be operational by 2027–2028, it will be the first center in France providing C-ion beam treatments, aiming to treat highly radioresistant cancers. The objectives are to build and validate the C400 machine and foster an industrial network around the manufacture and use of particle accelerators for therapeutic and diagnostic purposes. With the introduction of the C400 accelerator, many treatment perspectives will open, contributing to the growth and complexity of treatment options at CYCLHAD. By focusing on these advanced technologies, comprehensive research activities and future development plans, the CYCLHAD society and its scientific counterpart, ARCHADE, aim to become a pivotal center in the field of hadrontherapy, significantly impacting both clinical treatments and research advancements.

## Figures and Tables

**Table 1:** List of studies using particles within the ARCHADE network.

Type of Particle	Facility	Energy of Native Beam	LET of Particles (keV/µm)	Biological Model	Study ^(1)^	References
carbon ions	GANIL	95 MeV/A	28 and 73	CHS cell lines (SW1353, CH2879, OUMS27, L835)	RBE, cell cycle, g-H2AX	[10]
oxygen ions	GANIL	50 MeV/A	103	CHS cell line SW1353	Ki67, g-H2AX	[11]
carbon ions	GANIL	95 MeV/A	28, 50 and 100	GBM cell lines (U251-MG, GL15)	RBE, CFE, cell cycle, p-ERK	[12]
carbon ions	GANIL	75 MeV/A	34	Uveal melanoma (92.1, MEL270, SP6.5, MKT-BR, μ2, and TP17)	RBE, CFE, p-ERK	[13]
carbon ions	GANIL	95 MeV/A	75	NSCLC A549	CFE, cell cycle, gene analysis	[14]
carbon ions	GANIL	95 MeV/A	75	NSCLC A549	CFE, cell cycle, gene analysis, cytokines	[15]
carbon ions	GANIL	95 MeV/A	50	GBM (R633, TG1)	proliferation, cell cycle, gene analysis, cytokines, GSC	[16]
carbon ions	HIMAC	290 MeV/A	50	CHS (CH2879)	CFE, cell cycle, gene analysis, spheres, in vivo (mice)	[17]
carbon ions	GANIL	95 MeV/A	73	CHS (CH2879)	proliferation, CFE, WB PARPi	[18]
proton	INFN-LNS	62 MeV	11	CHS (CH2879)	proliferation, CFE, WB PARPi	[18]
carbon ions	GANIL	95 MeV/A	73	CHS cell lines (OUMS27, JJ012)	proliferation, CFE, gene analysis, WB PARPi	[19]
carbon ions	GANIL	95 MeV/A	28 and 73	GBM (U-251, T98G)	CFE, cell cycle, ELISA, ATMi, ATRi	[20]
proton	OCL	15.5 MeV	5; 42	GBM (U-251, T98G)	CFE, cell cycle, ELISA, ATMi, ATRi	[20]
carbon ions	GANIL	95 MeV/A	73	CHS cell lines (SW1353)	proliferation, CFE, MN, hyperspectral images	[21]
proton	IFIN-HH	18 MeV	12,6	CHS cell lines (SW1353)	CFE, MN, G-H2AX, Nano-P	[22]
proton	CYRCé	25 MeV	2–3	lymphocyte	circulating LY count	[23]
proton	CYRCé	25 MeV	2–3	lymphocyte	leucocyte interplay	[24]
carbon ions	GANIL	95 MeV/A	28	chondrocytes	CFE, WB, 3D senescence	[25]
carbon ions	GANIL	75 MeV/A	34	NHDF	CFE, comet, stress ox, cytokines	[26]
carbon ions	GANIL	75 MeV/A	34	NHDF	CFE, MN, 8-oxodG	[27]
proton	CPO	190 MeV	1.2	C57Bl/6 mice	survival, LY MN, SOD, LPO, cytokines	[28]
proton	CPO	190 MeV	1.2	C57Bl/6 mice	body weight, RNAseq	[29]
carbon ions	GANIL	95 MeV/A	28	GBM cell lines (U87-MG, T98G, LN18, M059K, M059J)	Spheres, WB, 8-oxo-dG, NRF2 KO cells	[30]
carbon ions	GANIL	95 MeV/A	28, 33	ADSCs	CFE, NRF2i, WB, differentiation	[31]
carbon ions	GANIL	95 MeV/A	28, 73	CHS (SW1353), chondrocytes	CFE, bystander medium transfer, MN, cytokines	[32]
carbon ions	HIMAC	290 MeV/A	50	CHS (SW1353), chondrocytes	CFE, bystander medium transfer, MN, cytokines	[32]
carbon ions	GANIL	95 MeV/A	73	CHS (SW1353), chondrocytes	mass spectrometry	[33]

^(1)^ CFE: colony forming efficiency, MN: micro-nuclei; WB: western blotting.

**Table 2:** Relative magnitudes of biological effects of carbon ions compared to X-rays in normal human skin fibroblasts for a variety of biological endpoint. Irradiation doses corresponding to 10% of survival defining a RBE of 3.28 for C-ions. OTM: Olive tail moment in comet assay, 8oxodGuo: 8-Oxo-2′-deoxyguanosine, SOD: superoxide dismutase, GPx: glutathione peroxidase, GSH/GSSG: ratio reduced glutathione/oxidized glutathione, TNF-α: tumor necrosis factor α, IL-6: interleukin 6. The methods used for these results are described in [26,27].

Cell survival	Clonogenicity	3.28
DNA damage	OTM	0.38
	Half time repair	0.31
	8-oxo-Gua	0.83
Protein damage	Carbonyls	0.21
lipid damage	peroxidation	0.49
Antioxidant enzymes	SOD	1.72
	Catalase	2.63
	GPx	1.69
Oxidative status	GSH/GSSG	0.48
Inflammation	TNF-a	0.79
	IL-6	0.74
